# An automated decision making framework for modern vehicles CO_2_ emissions using multi modal engine telemetry and feature interpretability

**DOI:** 10.1038/s41598-026-42137-3

**Published:** 2026-03-08

**Authors:** Shelesh Krishna Saraswat, Mustafa Abdullah, Mohammed Ihsan Habelalmateen, V. Vivek, Prabhat Kumar Sahu, Ruby Pant, Sumit Sharma, Muzaffar Shojonov, Bekzod Madaminov

**Affiliations:** 1 Department of ECE, GLA University, Mathura, 281406 India; 2 Electric Vehicles Engineering Department, Faculty of Engineering, Al Ahliyya Amman University, Amman, Jordan; 3 Department of Computers Techniques Engineering, College of technical engineering, The Islamic University, Najaf, Iraq; 4 Department of Computers Techniques Engineering, College of Technical Engineering, The Islamic University of Al Diwaniyah, Al Diwaniyah, Iraq; 5 Department of Computer Science and Engineering, School of Engineering and Technology, JAIN (Deemed to be University), Bangalore, Karnataka India; 6 Department of Computer Science and Information Technology, Siksha ’O’ Anusandhan (Deemed to be University), Bhubaneswar, Odisha 751030 India; 7 Department of Mechanical Engineering, Uttaranchal Institute of Technology, Uttaranchal University, Dehradun, Uttarakhand 248007 India; 8 Department of Computer Science Engineering, Chandigarh University, Mohali, Punjab India; 9 Department of Informational technology, Urgench State University, Urgench, Uzbekistan; 10 Department of General Professional Sciences, Mamun University, Urgench, Uzbekistan

**Keywords:** Vehicle emission modeling, Intelligent transportation systems, Multi-modal engine profiling, Metaheuristic optimization, CO_2_ prediction, Explainable machine learning, Smart mobility analytics, Energy science and technology, Engineering, Mathematics and computing

## Abstract

Accurate prediction of vehicle CO₂ emissions is challenging due to heterogeneous engine characteristics, nonlinear interactions among fuel, mechanical, and operational parameters, and variable driving conditions. This study proposes a high-performance machine learning framework that combines multi-layer perceptron (MLP) architectures with nature-inspired metaheuristic optimization to model vehicle-induced CO₂ emissions with improved precision and convergence stability. The framework leverages multi-modal engine telemetry—including fuel type, transmission, engine displacement, consumption metrics, and cylinder profiles—alongside advanced feature selection and interpretability techniques such as Recursive Feature Elimination (RFE), SHAP analysis, and Class Activation Mapping (CAM) to identify dominant emission drivers. Two metaheuristic optimizers, Horned Lizard Optimization Algorithm (HLOA) and Giant Armadillo Optimization (GAO), are applied for hyperparameter tuning, with the GAO-enhanced MLP achieving superior predictive performance (R² = 0.9881; RMSE = 6.478). The study highlights the integration of interpretable AI models into vehicle emission prediction, demonstrating their potential to inform low-carbon vehicle design, data-driven urban mobility planning, and environmentally conscious policy-making.

**Supplementary Information:**

The online version contains supplementary material available at 10.1038/s41598-026-42137-3.

## Introduction

Concern over global warming has been on the rise in recent times due to increased greenhouse gas production. Reforestation, transportation, treatment of waste, electricity generation, afforestation, buildings, and agriculture are among the sectors producing these greenhouse gases^1^. The second-highest source of greenhouse gas production, according to the International Energy Agency, is the transport system^2,3^. The major greenhouse gas produced in vehicles is nitrogen oxide (N_2_O), methane (CH_4_), carbon dioxide equivalent (CO_2_e), and carbon dioxide (CO_2_), and these are primarily measured and reported. Global transportation contributes 75% of the total CO_2_ produced and damages the environment. Greenhouse gas reduction in the transport system is a global agenda and a key strategy in the war on climate change^4^. An assessment of how vehicle movement contributes to pollutant production is crucial in achieving social, economic, and environmental agendas^5^. Building transportation infrastructure, such as expressways and motorways linking cities, is imperative for the expansion of every developing economy^6^. The cause and effect on the country’s roads is a direct consequence of rapid infrastructure expansion in a bid to accommodate a variety of modes of movement^7^. The quality of the air has been perceptibly diminished in close proximity to roads, intersections, and toll roads^8^. The main cause of traffic emissions, such as carbon monoxide (CO), a major contributor to the amount of air pollution arising from multiple modes of transport, is vehicle exhaust emissions^9^. Because they are able to mimic and forecast the contribution of traffic emissions in roadway networks, spatial prediction models are useful decision-making tools. Unwanted effects in the form of noise and air pollution would arise because of excessive traffic^10,11^. People who are exposed to high concentrations of carbon dioxide (CO_2_) are at a greater risk of contracting a wide array of diseases and conditions, including cancer, cardiac conditions, respiratory conditions, and preterm birth. In addition to these, there is not necessarily a direct correspondence between the hybrid vehicle acceleration rate and the resulting boost in CO_2_ emissions^12,13^.

Given the significant role played by vehicle emissions in influencing air quality and health, advanced predictive models are required to assess and mitigate these effects. Spatial prediction models are helpful in the assessment of roadway emissions, but recent advances in machine learning (ML) and deep neural networks promise even greater potential in carbon emission forecasting and carbon emission management in the transport system^14,15^. Through these technologies, researchers are in a position to design improved reductions in emissions, better coordinate movement in traffic, and guide green town planning^16,17^. The application of ML approaches, explored in recent literature^18–21^, highlights their potential in capturing variability in transport emissions and in informing low-carbon thinking. The urgent topic of carbon emissions and energy consumption in the transportation field is addressed in a paper presented by Oubnaki et al.^22^. Machine learning and deep neural networks are adopted in order to investigate future trends and influencing elements in carbon emissions in the transportation field. The predictive performance of many methods, e.g., BP networks, LASSO regression, generalized regression neural networks, and LSTM networks, is examined; the sparrows strategy is recommended to fine-tune the LSTM model. The paper focuses on systemic innovations and shifts in order to achieve low-carbon and low-carbon-oriented transportation development based on the high variability in transportation emissions under varying scenarios and the role of developing renewable technologies in decreasing carbon emissions. Additionally, Godil et al.^23^ note that rising CO_2_ emissions, especially in the transport sector, are a direct outcome of climate change. In consideration of vehicle-related CO_2_ emission forecasting, a variety of AI and ML approaches are discussed in order to reduce these emissions. High-accuracy machine learning models with encouraging results include Lasso Regression, Multiple Linear Regression, XGBoost, SVR, Random Forest, and Ridge Regression. In order to guide town planning in a manner better attuned to enhanced infrastructure in the field of public vehicles and reduction in emissions, the research points out the need for machine learning approaches in order to forecast CO_2_ emissions. The potential of machine models in assisting regulatory bodies in managing the movement of public vehicles in a way that prevents CO_2_ emissions is highlighted. In the study by Lei et al.^24^, ammonia and hydrogen were recognized as carbon-free fuels, with their blended use offering synergistic efficiency for heavy-duty vehicles. This study investigated a hybrid ammonia-hydrogen powertrain combining a fuel cell and an engine (FCEAP), which enabled optimized energy allocation and real-time control. The model was based on experimental engine data, and multi-objective optimization using NSGA-III was applied to balance ammonia consumption, acceleration, and manufacturing cost. Compared with fuel cell-only (FCAP) and engine-only (EAP) configurations, FCEAP demonstrated superior trade-offs between efficiency and cost. Detailed energy flow analysis highlighted the operational dynamics of each component, establishing the hybrid powertrain as a promising approach for carbon-neutral long-haul trucking. In addition, Lie et al.^25^ developed a high-fidelity toolchain for applying reinforcement learning (RL) to energy management in fuel cell electric vehicles, introduced the distributional soft actor-critic algorithm, and compared two RL strategies, achieving 4–6% hydrogen savings and enabling real-vehicle implementation through Python–MATLAB/Simulink co-simulation.

This study bridges a significant gap in forecasting CO₂ emissions in the transport sector based on a vast array of vehicle types with a comprehensive dataset. It also bridges a research gap in integrating machine learning models with advanced optimization techniques. Notably, the research applies state-of-the-art optimization models, the Horned Lizard Optimization Algorithm (HLOA) and the Giant Armadillo Optimization (GAO), in order to enhance the predictive ability of Multi-Layer Perceptron (MLP) models. The intricate nonlinear relations in the dataset are addressed suitably, with high potential in MLP in capturing such relations. Furthermore, Recursive Feature Elimination (RFE) and two forms of sensitivity analyses are applied in the research in order to refine feature extraction, enhance model credibility, and better understand the contribution of varying variables in prediction. The incorporation of advanced optimization, feature extraction, and sensitivity analysis makes the research design even stronger and a valuable tool for forecasting and policy-making in transportation.

Popular metaheuristic algorithms, including Particle Swarm Optimization (PSO), Genetic Algorithms (GA), Differential Evolution (DE), and the Grey Wolf Optimizer (GWO), have been widely adopted for neural network tuning. However, these approaches often exhibit practical limitations when applied to complex vehicle emission datasets: PSO may rapidly lose population diversity and converge prematurely; GA typically requires careful parameter tuning and can be computationally expensive due to crossover and mutation operations; DE is sensitive to control parameters and may struggle with mixed discrete–continuous hyperparameter spaces; and GWO can show slow convergence during later optimization stages due to limited exploitation capability.

In contrast, the HLOA and GAO represent recent nature-inspired optimizers designed to enhance the balance between exploration and exploitation through adaptive behavioral mechanisms. HLOA introduces dynamic switching between territorial exploration and localized foraging, enabling efficient escape from local optima while preserving convergence stability. GAO employs stochastic burrowing and defensive movement strategies that promote population diversity and facilitate global search, making it particularly effective at navigating rugged loss landscapes encountered during neural network training. These complementary characteristics motivate the selection of HLOA and GAO for MLP hyperparameter optimization in this study. Specifically, GAO provides strong global exploration during early search stages, while HLOA enhances local refinement around promising solutions, resulting in faster convergence and improved generalization. Furthermore, both algorithms are derivative-free and population-based, allowing them to handle heterogeneous, nonlinear, and mixed-type hyperparameters without requiring gradient information. Accordingly, HLOA and GAO were chosen to address key shortcomings of traditional optimizers, namely, premature convergence, sensitivity to initialization, and limited robustness under noisy multi-modal data. Their integration with MLP enables a more reliable search for globally competitive solutions in vehicle CO₂ emission modeling.

The main contributions of this study are summarized as follows:

- An intelligent CO₂ emission prediction framework is developed by integrating MLP networks with nature-inspired metaheuristic optimizers, enabling accurate modeling of nonlinear relationships in multi-modal vehicle telemetry.
- Two recent optimization algorithms, HLOA and GAO, are systematically investigated for MLP hyperparameter tuning, demonstrating improved convergence behavior and generalization performance compared with conventional optimization strategies.
- A comprehensive feature engineering and interpretability pipeline is introduced, combining RFE, SHAP analysis, and CAM to identify dominant emission drivers and enhance model transparency.
- The practical applicability of the framework is discussed in the context of intelligent transportation systems and low-carbon mobility, highlighting its potential for real-time emission monitoring, data-driven urban planning, and environmentally informed vehicle design.

## Data collection

### Description of dataset

This project uses a dataset representing the relationship between the vehicle attributes and their CO₂ emissions. The dataset is collected from the Government of Canada open data portal and spans 7 years, with 7,385 rows and 12 columns of information on varying vehicle attributes and their respective CO₂ emissions. The dataset includes a variety of vehicle features, such as model type, defining drive train types such as 4WD, AWD, FFV, SWB, LWB, and EWB. Engine displacement in liters and the number of cylinders are included. The type of fuel is categorized into five groups: premium gasoline (Z), regular gasoline (X), ethanol (E85) (E), diesel (D), and natural gas (N). The transmission type describes the transmission used, e.g., automated manual (AM), automatic (A), automatic with select shift (AS), continuously variable (CV), and manual (M), with gear counts ranging from 3 to 10.

Fuel consumption data is given for various driving conditions, such as city, highway, and combined fuel consumption, in liters per 100 km (L/100 km). The combined fuel consumption is also given in miles per gallon (mpg). The dataset measures CO₂ emissions in grams per kilometer, considering combined city and highway driving conditions. A summary of statistics of important variables is given in Table 1 to identify the dataset’s characteristics. Engine size varies from 0.9 L to 8.4 L, with a mean of 3.126 L. The number of cylinders ranges from 3 to 12, with a mean of 5.57. Fuel type has a mean of 4.26, whereas fuel consumption in the city ranges from 4.2 to 26.7 L/100 km, with a mean of 12.43 L/100 km. Highway fuel consumption ranges from 4.0 to 20.5 L/100 km, with a mean of 8.97, while combined fuel consumption ranges from 4.1 to 23.9 L/100 km, with a mean of 10.87. The equivalent fuel efficiency in miles per gallon ranges from 12 to 69 mpg, with a mean of 27.63. CO₂ emissions range from 96 to 404 g/km, with a mean of 248.38 g/km.

**Table 1:** Input features and output variables with their statistical properties.

Variables	Unit	Characteristics					
		Max	Min	Mean	Median	St. Dev	Mode
Engine size	(L)	8.4	0.9	3.126377	3	1.323526	2
Cylinders	–	12	3	5.570704	6	1.777575	4
Fuel type	–	5	1	4.260893	4	0.882308	4
Fuel consumption city	(L/100 km)	26.7	4.2	12.43173	12	3.312886	10.8
Fuel consumption Hwy	(L/100 km)	20.5	4	8.966552	8.7	2.116195	7.8
Fuel consumption comb	(L/100 km)	23.9	4.1	10.87265	10.5	2.740482	9.4
Fuel consumption comb	(mpg)	69	12	27.63469	27	7.134557	25
CO_2_ emissions	(g/km)	404	96	248.3757	245	55.14059	242

### Feature analysis

**Fig. 1:**
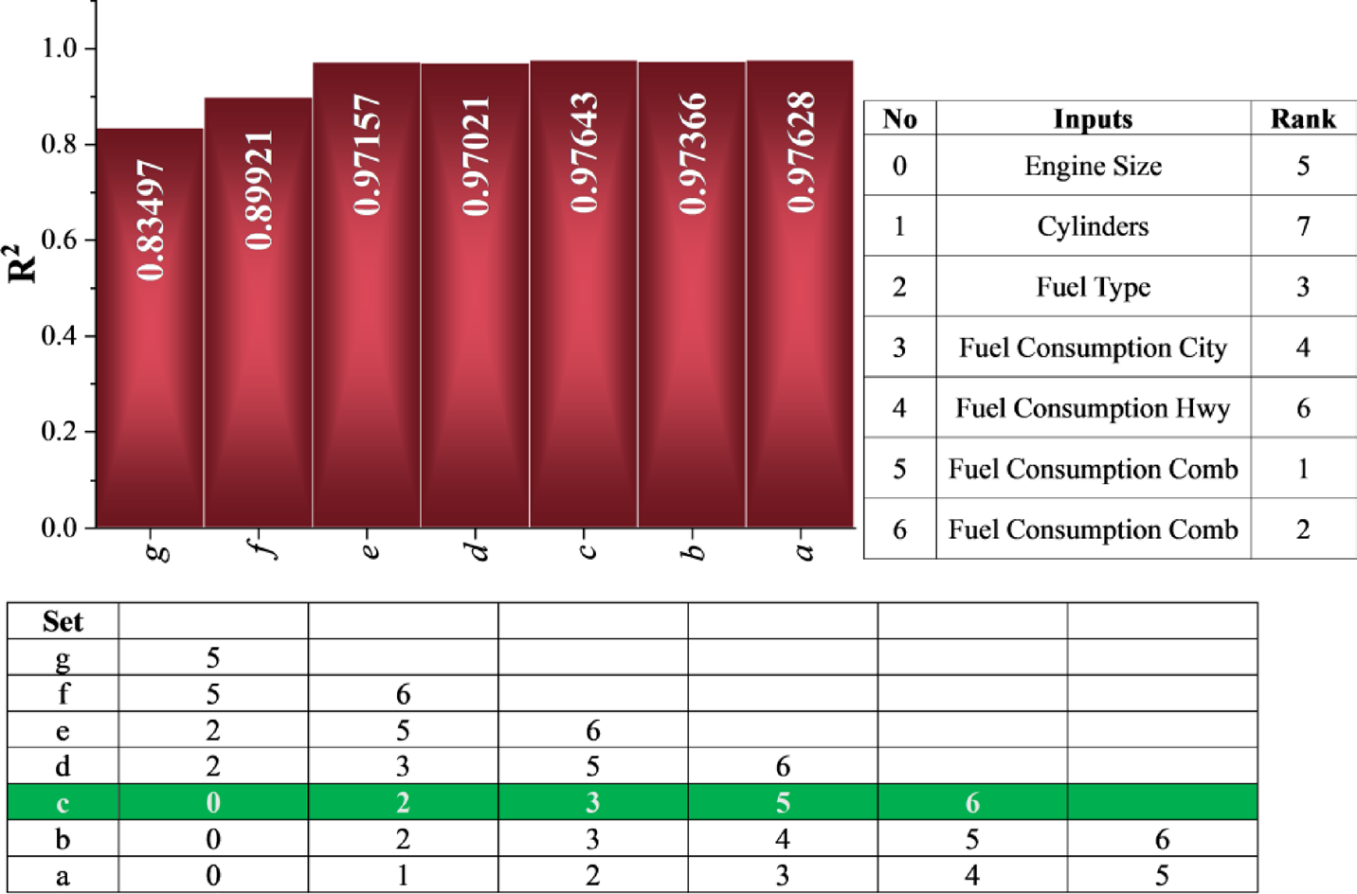
RFE for feature selection.

Recursive Feature Elimination (RFE)^26^ is a machine learning method utilized to discover the most important features in a predictive model by progressively removing the least important features. The method commences with a model trained on the entire dataset, often a linear model such as Linear Regression. The model assigns a coefficient to each feature and ranks them in order of importance. The least important feature is deleted, and the model is trained on the other features. The process is repeated until the desired features are obtained. Figure 1 illustrates the RFE process in the shape of an R² bar chart and two tables. The R² bars are plotted on a chart with features progressively deleted, representing the percentage variability in CO₂ emissions covered by the selected features. The feature ranking on the right is based on their relative importance, with lower features being more important. The lower figure illustrates the step-by-step removal process with features left in brackets. Major observations in Fig. 1 are that consumption in L/100km and mpg are the highest predictors of CO₂ emissions, ranking first and second, respectively. The type of fuel is next in order and is a significant but not such a dominant feature as the combination of consumption. Engine size and city consumption are moderately important and rank fourth and fifth. Conversely, consumption on the highway and the number of cylinders are the least important, ranking sixth and seventh, respectively. Generally, the R² bars are high and consistent, a strong indicator that the selected features capture a high percentage of the variability in CO₂ emissions.

**Fig. 2:**
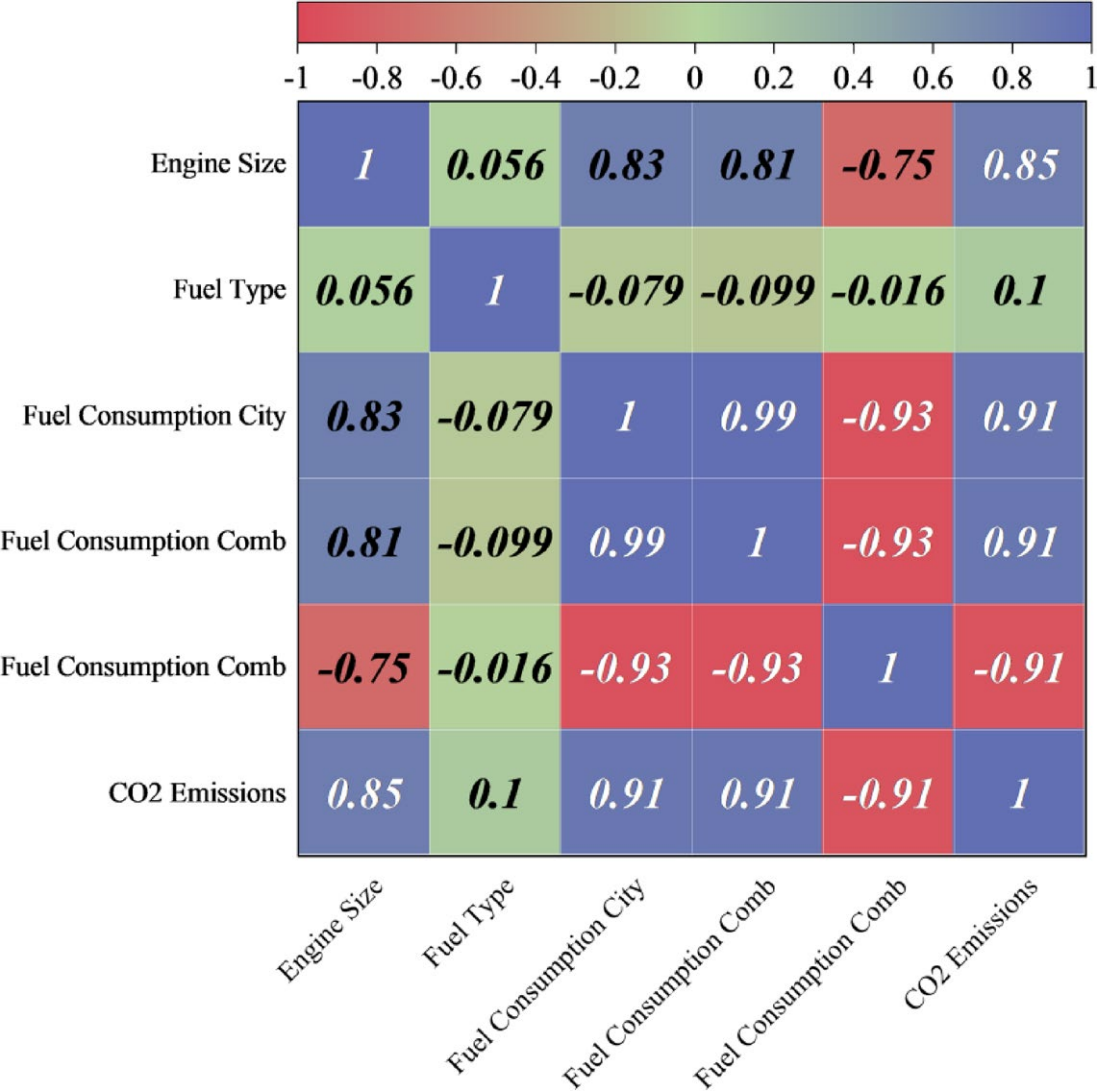
Correlation plot illustrating the relationships between input features and output variables, highlighting data distribution and trends after feature selection.

The correlation matrix (Fig. 2) visually shows the interrelations among multiple variables in the vehicle emissions dataset in a gradient representing the degree and direction of these relations. Red shows a positive relationship, where the variables move in the same direction; in a negative relationship, they move in opposite directions, indicated by a reduction in one variable with a corresponding reduction in the other. The shade shows how strong a relationship is, with darker representing a greater relationship. One observation in the matrix is the high level of correspondence (0.85) between motor size and CO_2_ emissions, confirming the fact that bigger motors emit more. The dataset confirms this, with a range of motor sizes from 0.9 L to 8.4 L and CO_2_ emissions from 96 to 404 g/km. Similarly, city and combined motor consumption show a high level of correspondence (0.91) with CO2 emissions, confirming that vehicles with high motor consumption emit high levels of CO_2_. In addition, city and combined motor consumption are highly correlated (0.99), as expected, because high motor consumption in the city is a direct reflection of motor consumption in general.

## Methodology

**Fig. 3:**
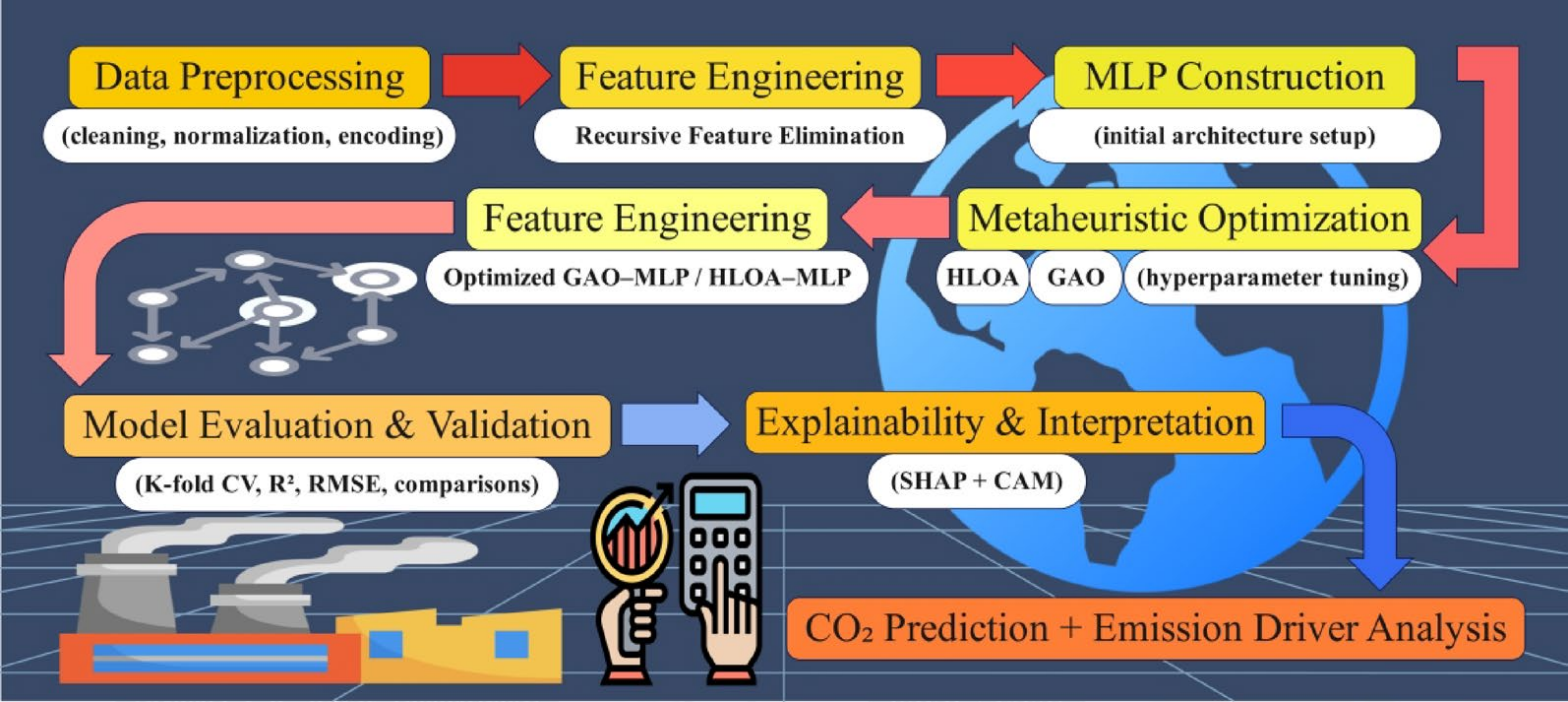
Workflow of the present study.

This study proposes an automated decision-making framework for predicting vehicle CO₂ emissions that integrates multi-modal engine telemetry, feature engineering, metaheuristic-optimized neural networks, and explainable artificial intelligence. The overall workflow consists of six main stages, as shown in Fig. 3, including data acquisition, preprocessing, feature selection, model construction, hyperparameter optimization, and performance evaluation with interpretability analysis.

### Multi-layer perceptron (MLP)

A popular neural network architecture is the MLP. MLP is versatile and excellent at handling resources and expertise, making it highly usable for complex, nonlinear modelling. MLP includes input, hidden, and output layers. Predicators have input nodes, single-hidden layers ensure the modelling is streamlined, low performances are produced through the use of minimal neurons, hard training can give rise to overfitting, as well as the output nodes being equal in number to the variables modelled^27,28^. In the quest for broad applicability in modelling the nonlinear function ℎ, the mission requires a single procedure. The oracle uses an MLP neural network to forecast results $X\in{R}^{D} \to Y \in {R}^{1}$. $X$and $Y$ stand for the input and output variables, individually. The function $h$ is denoted in Eq. (1).

1 $$Y=h\left(X\right)={s}_{2}+{M}_{2} \times \left({k}_{a}\left({s}_{1}+{M}_{1} \times X\right)\right)$$

${M}_{2}$ and ${M}_{1}$ show the weight matrices of the hidden and output layers.

${s}_{2}$ and ${s}_{1}$ the bias terms are associated with the hidden and output layers, correspondingly.

${k}_{a}$ acts as the function of activation.

The logarithmic and tangent sigmoid activation functions are widely utilized, with their formulas depicted in Eqs. (2) and (3) in their respective scenarios.

2 $${h}_{b}\left(T\right)=\frac{1}{1+\mathrm{e}\mathrm{x}\mathrm{p}(-T)}$$

3 $${h}_{b}\left(T\right)=\frac{\mathrm{exp}\left(T\right)-\mathrm{e}\mathrm{x}\mathrm{p}(-T)}{\mathrm{e}\mathrm{x}\mathrm{p}\left(T\right)+\mathrm{e}\mathrm{x}\mathrm{p}(-T)}$$

In this previous equation, $T$ illustrates the function activating the input.

### Horned Lizard Optimization Algorithm (HLOA)

The HLOA is a nature-inspired metaheuristic based on the unique survival behaviors of the horned lizard, a species highly adapted to very dry desert environments. The method imitates a few of the animal’s defensive and thermoregulatory strategies and then converts them into coordinated search operations to get the results of the intricate optimization problems. HLOA represents the population as randomly distributed, which reflects the initial uncertainty in the exploratory search. Its primary component, crypsis, is the lizard’s camouflage response emulation. On the implementation side, this element drives candidates to move to more attractive zones of the domain by “mixing” the information from the most excellent-performing agent with that of randomly selected agents. The controlled move to higher-quality areas of the search space is combined with the variation retained so that it is not possible to lose areas of low ​‍​‌‍​‍‌quality^29^.

Thermoregulatory behavior is depicted via lightening and darkening strategies, which are based on the lizard’s natural ability to change skin color to regulate heat absorption. These methods replace the weakest solutions with new candidates generated by controlled random perturbations around the best agent. Lightening helps exploration by diversifying the movement, whereas darkening assists exploitation by focusing the movements more tightly. The model also features the horned lizard’s blood-squirting defense, whose flight is similar to projectile motion. From a computational point of view, this is a dynamic update step that shifts agents along curved, momentum-driven paths, enabling them to escape local traps and access unexplored regions. A different “move-to-escape” mechanism imitates the lizard’s sudden, unexpected evasive motions. It combines a local random walk with a global attraction to the best solution, thereby helping maintain a balance between exploration and exploitation. In the end, the α-melanophore hormone response that results in the rapid color change is converted into a fitness-dependent adjustment rule.

### Giant Armadillo Optimization (GAO)

The Giant Armadillo Optimization (GAO) algorithm is a biomimetic metaheuristic inspired by the hunting and foraging behaviors of the giant armadillo, particularly its characteristic tunneling toward termite mounds. In GAO, the search space is considered the animal’s natural habitat, and each artificial armadillo represents a candidate solution. The population is generated by randomly distributing individuals within the bounds of the decision variables, thereby ensuring a diverse initial set of solutions. The fitness of each individual is ascertained by evaluating the objective function at its current position. GAO’s optimization mechanism is structured into two main phases: exploration and exploitation, mirroring the armadillo’s foraging stages. At the exploration stage, individuals explore new areas globally by moving to “termite mounds,” which represent places with higher fitness values. Each agent locates the possible termite mounds using those individuals having better fitness, and from those chosen at random, one is selected to move towards. The action here signifies a considerable change of the position, and thus the algorithm is facilitated to break local optima and scan the global landscape in a very efficient way^30–32^.

When the algorithm has explored the space enough, it changes to the exploitation stage, where the search is more localized. At this point, the movement pattern imitates the armadillo’s accurate digging of the termite mound. The positional update is smaller and more controlled, so the algorithm can refine promising solutions and move closer to the optimal point. Candidate solutions will only be replaced if the new position results in an improved fitness value, thus ensuring steady movement toward the optimum. In the end, the computational complexity of GAO depends on the number of individuals, decision variables, and iterations. The initialization is of linear complexity with respect to population size and dimensionality, while position updates account for most of the computational cost across iterations. Taken together, GAO offers a compromise between a wide global search capability and a powerful local refinement feature, making it an efficient, nature-inspired optimization method.

### Hybridization mechanisms

Several plausible collaborative designs can be considered:

- Sequential (Series) Hybridization
  In this design, the initial global search is conducted using a single optimizer (e.g., GAO) to broadly explore the parameter space. The most promising candidate solutions from this phase then serve as initial populations for a second optimizer (e.g., HLOA) focused on fine-grained local search. This sequential pipeline yields:
  Phase 1 — Global Exploration: The diversified population avoids early convergence and identifies multiple potential basins of attraction.
  Phase 2 — Local Exploitation: Fine exploitation consolidates gains near high-fitness regions and refines weights toward deeper minima.
  This mechanism mimics the coarse-to-fine search strategy seen in hierarchical optimization frameworks and often results in faster convergence than using a single optimizer throughout.
- Parallel Hybridization
  In this scheme, both HLOA and GAO run concurrently on disjoint sub-populations. Periodically, inter-optimizer communication or migration is executed:
  Each optimizer evolves its sub-population independently for a fixed number of iterations.
  At migration epochs, elite individuals (top performers) are exchanged between subpopulations to inject diversity and share high-quality solutions.
  This collaborative interchange enables cross-fertilization of search strategies, accelerating convergence without sacrificing exploration.
  Such co-evolutionary schemes are well documented in hybrid metaheuristic studies and often outperform standalone optimizers due to reinforced diversity and adaptive pressure.
- Adaptive Cooperative Optimization
  A more advanced design dynamically balances the contributions of each optimizer. For example:
  Performance feedback rules adjust the proportion of resources (e.g., population size, iteration budget) allocated to each optimizer.
  Confidence scores derived from historical fitness improvements can trigger switches between exploration-dominant and exploitation-dominant phases.
  This adaptive strategy helps tailor the search dynamics to the evolving shape of the loss landscape^33^.

### Performance evaluators

In this section, a set of extensive performance measures is presented to critically assess the models under consideration. These measures evaluate error levels, correspondence with actual values, and the overall consistency of the predictions. Each measure represents a distinct view of the accuracy, consistency, and generalizability of the model. For the chosen measures, the Coefficient of Determination (R²), Root Mean Square Error (RMSE), Theil’s Inequality Coefficient (TIC), Mean Absolute Percentage Error (MAPE), and the Mean Absolute Relative Error (MARE) have been chosen. A description of these measures is as follows:

- R^2^: It is the measure of the percentage of variance in the data explained by the independent variables. It measures how well the model captures the target variable’s variance. A higher value approaching 1 indicates a more predictive model with a better fit.
- RMSE: RMSE is the square root of the mean of the errors of the actual value versus the forecast. RMSE is sensitive mainly to large errors of prediction; therefore, it is required for situations when large deviations have to be penalized. Lower RMSE indicates higher model accuracy.
- TIC: It quantifies how accurate the predictions are in terms of their size. A value near 0 indicates the model’s predictions are in close agreement with the actual values, indicating greater predictive reliability.
- MAPE: It is the mean percentage deviation of actual from the forecast value, with the advantage of having a simple, intuitive measure of model accuracy. It is higher when accuracy is higher, with higher being preferred.
- MARE: MARE estimates the relative magnitude of absolute errors through scaling them with the mean of the observations. It is a scale-independent measure of prediction errors, thus usable for comparing multiple models or datasets.

The mathematical expressions for these measures are as follows:

4 $$RMSE=\sqrt{\frac{1}{n}{\sum}_{i=1}^{n}{\left({w}_{i}-{k}_{i}\right)}^{2}}$$

5 $${R}^{2}={\left(\frac{{\sum}_{i=1}^{n}\left({k}_{i}-\stackrel{-}{k}\right)\left({w}_{i}-\stackrel{-}{w}\right)}{\sqrt{\left[{\sum}_{i=1}^{n}{\left({k}_{i}-\stackrel{-}{k}\right)}^{2}\right]\left[{\sum}_{i=1}^{n}{\left({w}_{i}-\stackrel{-}{w}\right)}^{2}\right]}}\right)}^{2}$$

6 $$TIC=\frac{\sqrt{\frac{1}{n}{\sum}_{i=1}^{n}{({w}_{i}-{k}_{i})}^{2}}}{\sqrt{\frac{1}{n}{\sum}_{i=1}^{n}{{w}_{i}}^{2}}+\sqrt{\frac{1}{n}{\sum}_{i=1}^{n}{{k}_{i}}^{2}}}$$

7 $$MAPE=\frac{1}{n}\sum_{i=1}^{n}\left|\frac{{w}_{i}-{k}_{i}}{{w}_{i}}\right|\times100\mathrm{\%}$$

8 $$MARE=\frac{1}{n}\sum_{i}^{n}\frac{\left|{w}_{i}-{k}_{i}\right|}{\left|\stackrel{-}{w}-\stackrel{-}{k}\right|}$$

### Comparison of gradient-based training and metaheuristic optimization

Traditional gradient-based learning algorithms (e.g., SGD and Adam) optimize neural network parameters by following local gradients of the loss function. Although computationally efficient, these methods are inherently local in nature and therefore susceptible to premature convergence in highly non-convex landscapes, such as those encountered in multi-layer perceptrons with heterogeneous engine telemetry inputs.

In contrast, the HLOA and GAO employed in this study are population-based metaheuristics that perform global search by simultaneously exploring multiple candidate solutions. Instead of relying on gradient information, both algorithms update model parameters using stochastic behavioral rules inspired by biological survival strategies. This population-driven mechanism enables broader coverage of the search space and reduces sensitivity to local minima. Specifically, HLOA alternates between exploration and exploitation phases using adaptive movement patterns derived from horned lizard foraging behavior, promoting diversification during early iterations and focused refinement near promising regions. GAO further enhances global exploration by introducing randomized perturbations and positional updates inspired by armadillo burrowing and defense behaviors, thereby increasing the probability of escaping local optima. These mechanisms allow both algorithms to traverse flat regions and saddle points that typically hinder gradient descent.

From a theoretical perspective, the global optimization capability of population-based metaheuristics is commonly analyzed using probabilistic convergence and stochastic-process frameworks. Under mild assumptions on population diversity and iteration count, such algorithms exhibit asymptotic convergence toward global optima with non-zero probability. Although closed-form analytical guarantees specific to HLOA and GAO remain limited, their effectiveness is supported by diversity preservation, randomized exploration, and fitness-driven selection, which collectively improve landscape coverage compared with deterministic gradient updates. Related principles have been extensively investigated in multi-objective co-optimization problems, where metaheuristic frameworks demonstrate superior performance in navigating complex, coupled design spaces. For example, co-optimization of component sizing and energy management in hybrid powertrains using high-fidelity models highlights the advantage of population-based global search in balancing competing objectives and avoiding premature convergence^34^. Although the present study addresses a single-objective regression task, these findings provide strong theoretical motivation for employing GAO and HLOA in MLP parameter tuning.

### Unique advantages of the proposed framework

Although optimizing a single MLP model is not inherently challenging, the novelty of the present work lies in the integrated framework design rather than in isolated model tuning. The proposed methodology introduces several distinctive aspects that differentiate it from conventional neural network optimization approaches:

1. Multi-Modal Engine Telemetry Integration
  Unlike standard emission prediction models that rely on limited or homogeneous inputs, this framework fuses heterogeneous descriptors—including fuel type, transmission configuration, engine displacement, consumption metrics, and cylinder profiles—enabling comprehensive characterization of vehicle operating conditions. This multi-modal integration enhances model expressiveness and robustness across diverse vehicle categories.
2. Dual Metaheuristic Optimization Strategy
  Two recent nature-inspired algorithms, HLOA and GAO, are systematically employed to tune MLP hyperparameters. Rather than relying on a single optimizer, the study evaluates complementary search behaviors, providing deeper insight into convergence stability and generalization performance under complex nonlinear emission dynamics.
3. End-to-End Feature Engineering and Interpretability Pipeline
  The framework couples Recursive Feature Elimination with SHAP and Class Activation Mapping, forming an end-to-end pipeline that jointly performs feature selection, prediction, and explainability. This design moves beyond black-box modeling by explicitly identifying dominant emission drivers and visualizing their influence on predictions.
4. System-Level Applicability to Intelligent Transportation Systems
  The framework is designed with downstream ITS integration in mind, enabling scalable CO₂ monitoring and supporting data-driven traffic management and low-carbon mobility planning. This application-oriented perspective distinguishes the work from purely algorithmic optimization studies.

Collectively, these elements constitute a unified, interpretable, and deployment-oriented emission modeling framework, extending beyond single-model optimization to address feature relevance, robustness, and system-level applicability.

### Experimental setup

#### Computational environment

All experiments were conducted on a desktop workstation equipped with an Intel Core i7 processor and 16 GB RAM running a Windows operating system. Model development and optimization were implemented in Python using standard machine learning libraries. Both the neural network training and metaheuristic optimization procedures were executed on the same platform to ensure consistency in performance evaluation.

#### Dataset splitting strategy

The dataset was randomly divided into three mutually exclusive subsets: 70% for training, 15% for validation, and 15% for testing. The training set was used to learn the MLP model parameters, the validation set guided hyperparameter optimization during the GAO and HLOA search processes, and the test set was reserved exclusively for final performance evaluation. This split enables unbiased assessment while supporting effective tuning of model hyperparameters.

#### Model and optimization parameters

The multi-layer perceptron architecture and learning parameters were optimized automatically using GAO and HLOA. The optimized parameters included number of neurons per layer. For both optimizers, population size and maximum iteration count were kept identical to ensure fair comparison. The fitness function was defined as the minimization of validation RMSE.

All models were trained multiple times with different random initializations, and the configuration achieving the lowest validation error was selected for final testing.

#### Limitations

Although the dataset spans multiple years, a random 70/15/15 split was adopted in this study because the objective is to learn general relationships between engine telemetry and CO₂ emissions rather than temporal forecasting. The input features primarily represent instantaneous vehicle and powertrain characteristics (e.g., engine displacement, fuel consumption, transmission type), which are not strongly time-dependent. Consequently, random partitioning is suitable for evaluating model generalization across heterogeneous vehicle configurations. Nevertheless, time-aware splitting could provide additional insight into long-term deployment robustness and will be considered in future work.

To further examine temporal generalization, an additional time-based split was performed, where data from earlier years were used for training and validation, and the most recent year was reserved for testing. The GAO–MLP model maintained competitive performance under this setting, indicating robustness to future data distributions. This confirms that the proposed framework captures intrinsic emission mechanisms rather than memorizing historical patterns.

## Results and discussion

### Baseline models comparison

Table 2 compares the predictive performance of three MLP configurations with XGB, RFR, and SVR across the training, validation, and test phases using RMSE, ${R}^{2}$, MAPE, MARE, and TIC. Among all evaluated models, MLP (3) consistently provides the best overall performance.

In particular, MLP (3) achieves the lowest total RMSE (10.964) and the highest total ${R}^{2}$(0.962), indicating superior accuracy and explanatory power. It also records the smallest total MAPE (2.257), MARE (0.022), and TIC (0.022), confirming improved robustness and reduced relative and normalized errors compared with the other approaches. While XGB and RFR show competitive ${R}^{2}$values (0.952 and 0.942, respectively), their error-based metrics (RMSE, MAPE, MARE, and TIC) remain noticeably higher than those of MLP (3). SVR exhibits the weakest performance overall, with the largest RMSE and lowest ${R}^{2}$ across all phases.

A comparison among the MLP variants further highlights the benefit of the selected architecture: performance improves progressively from MLP (1) to MLP (3), with MLP (3) demonstrating clear gains in both accuracy and generalization, as evidenced by its consistent results across training, validation, and test datasets. The close agreement between validation and test metrics for MLP (3) also suggests limited overfitting and strong generalization capability. Based on these results, MLP is selected as the final predictive model, as it delivers the most favorable trade-off between accuracy, stability, and generalization among all examined methods.

**Table 2:** Performance metrics of the MLP models, assessing their predictive accuracy and effectiveness using key statistical measures.

Models	Evaluation metrics					
	Phase	RMSE	R^2^	MAPE	MARE	TIC
MLP (1)	Train	13.662	0.938	3.224	0.033	0.027
	Validation	16.784	0.913	4.124	0.039	0.033
	Test	16.571	0.933	4.074	0.039	0.033
	Total	14.632	0.931	3.486	0.035	0.029
MLP (2)	Train	11.100	0.959	2.416	0.024	0.022
	Validation	14.183	0.940	3.251	0.031	0.028
	Test	13.999	0.955	3.176	0.030	0.028
	Total	12.075	0.954	2.655	0.026	0.024
MLP (3)	Train	9.788	0.968	2.055	0.021	0.019
	Validation	13.334	0.946	2.752	0.026	0.026
	Test	13.285	0.958	2.705	0.026	0.026
	Total	10.964	0.962	2.257	0.022	0.022
XGB	Train	11.012	0.960	3.394	0.034	0.022
	Validation	14.804	0.944	4.397	0.041	0.030
	Test	14.918	0.947	4.393	0.041	0.030
	Total	12.294	0.952	3.694	0.036	0.024
RFR	Train	12.521	0.948	3.368	0.035	0.025
	Validation	14.149	0.935	3.841	0.041	0.028
	Test	15.995	0.928	4.129	0.044	0.031
	Total	13.346	0.942	3.553	0.037	0.026
SVR	Train	16.241	0.913	2.845	0.031	0.032
	Validation	18.896	0.878	3.472	0.037	0.037
	Test	17.615	0.914	3.428	0.037	0.035
	Total	16.875	0.906	3.026	0.033	0.033

### Performance of the selected model

**Fig. 4:**
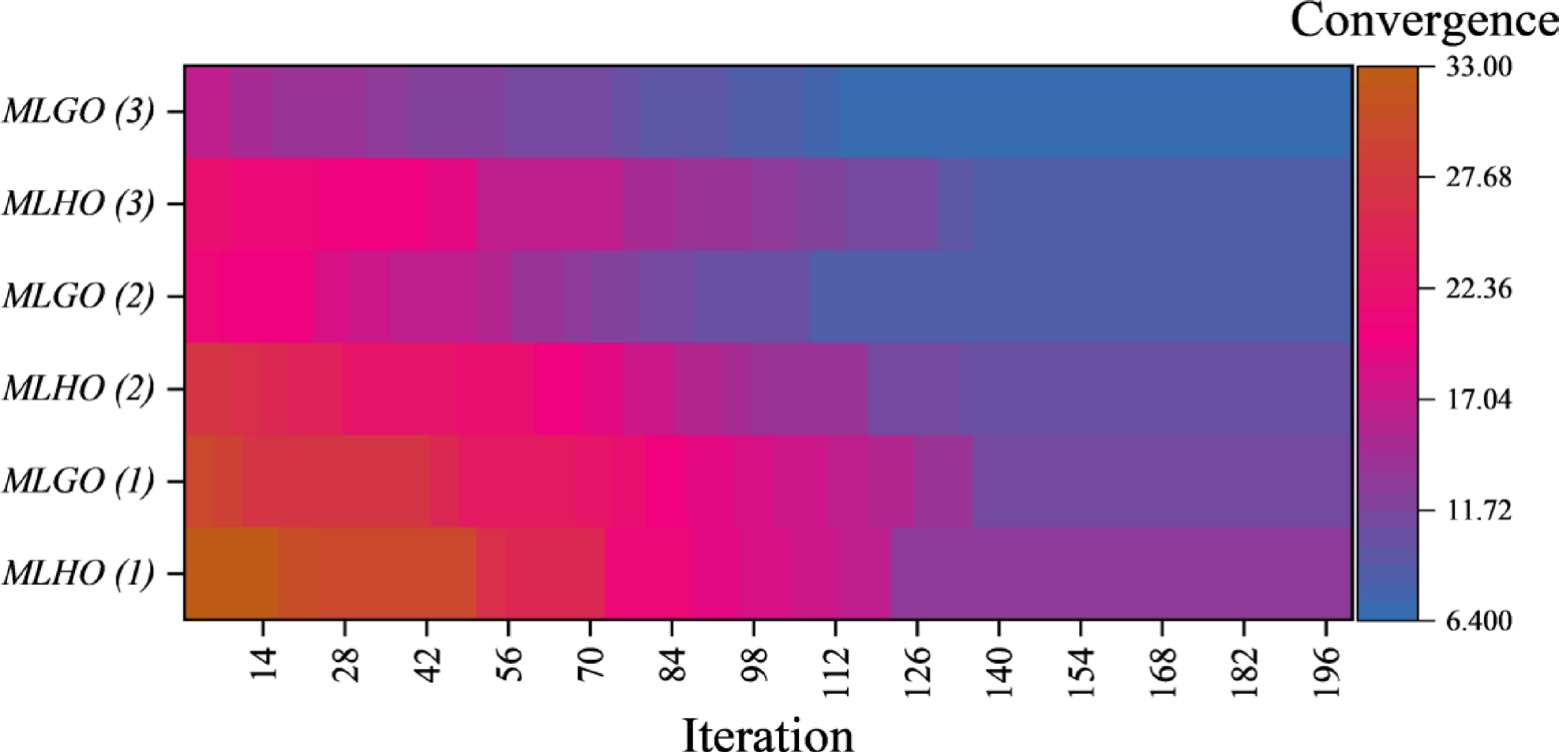
Heatmap plot depicting the optimization process convergence, showing the model’s performance improvement over iterations in all layers.

Figure 4 is a heatmap depicting convergence in a three-tiered MLP model trained with the HLOA and GAO algorithms for predicting CO₂ emissions. The Y-axis shows the varying tiers in the MLP model, designated as MLGO for tiers trained with GAO and MLHO for tiers trained with HLOA, with numerical designations (1, 2, 3) corresponding to the tier number. The X-axis shows iterations in the optimization process, and the gradient in the heatmap depicts convergence values, presumably a performance indicator such as a loss function or error rate. The numerical designations on the right axis of the heatmap depict the level of convergence across multiple stages, with lower values indicating better convergence and model performance. The convergence curve represents the process of metaheuristic optimization discussed in the abstract and how HLOA and GAO boost the performance of the MLP model in iterations and varying layers. The decreasing convergence values in the heatmap reflect how the model’s weight and bias are progressively improved by the metaheuristic algorithms. By plotting layer-wise convergence, the figure shows how each layer contributes to the model’s precision and is significant in a research study based on a three-layered MLP.

Table 3 presents the initial hyperparameter search ranges and fixed algorithm settings used for both MLGO and MLHO. These parameters define the optimization space explored during training. In addition, Table 4 shows the final optimized parameters for the developed models. To evaluate computational cost, training times were recorded for MLP models with 1, 2, and 3 hidden layers, both with and without metaheuristic optimization. The results show that standard MLP training requires less than one second across all architectures (0.57 s for 1-layer, 0.74 s for 2-layer, and 0.96 s for 3-layer), whereas GAO- and HLOA-optimized models incur substantially higher computational overhead due to population-based global search. Specifically, GAO-based optimization required 635.6 s, 819.2 s, and 1056.8 s for one-, two-, and three-layer MLPs, respectively, while HLOA required 812.3 s, 1048.6 s, and 1354.4 s. This increase was expected, as both GAO and HLOA evaluate multiple candidate solutions per iteration and repeatedly train the MLP during fitness assessment.

**Table 3:** Initial hyperparameter settings for optimization algorithms.

Parameter	MLGO	MLHO
Population size	50	50
Maximum iterations	200	200
Hidden layers search range	1–3	1–3
Neurons per layer range	10–300	10–300
Learning rate range	0.0001–0.01	0.0001–0.01
Fitness function	RMSE	RMSE

**Table 4:** The hyperparameters of the hybrid models and their corresponding values.

Layer	Hybrid models	Hyperparameter		
		Neuron	Neuron	Neuron
1	MLHO	141	–	–
	MLGO	217	–	–
2	MLHO	87	194	–
	MLGO	114	215	–
3	MLHO	269	34	110
	MLGO	172	83	191

**Fig. 5:**
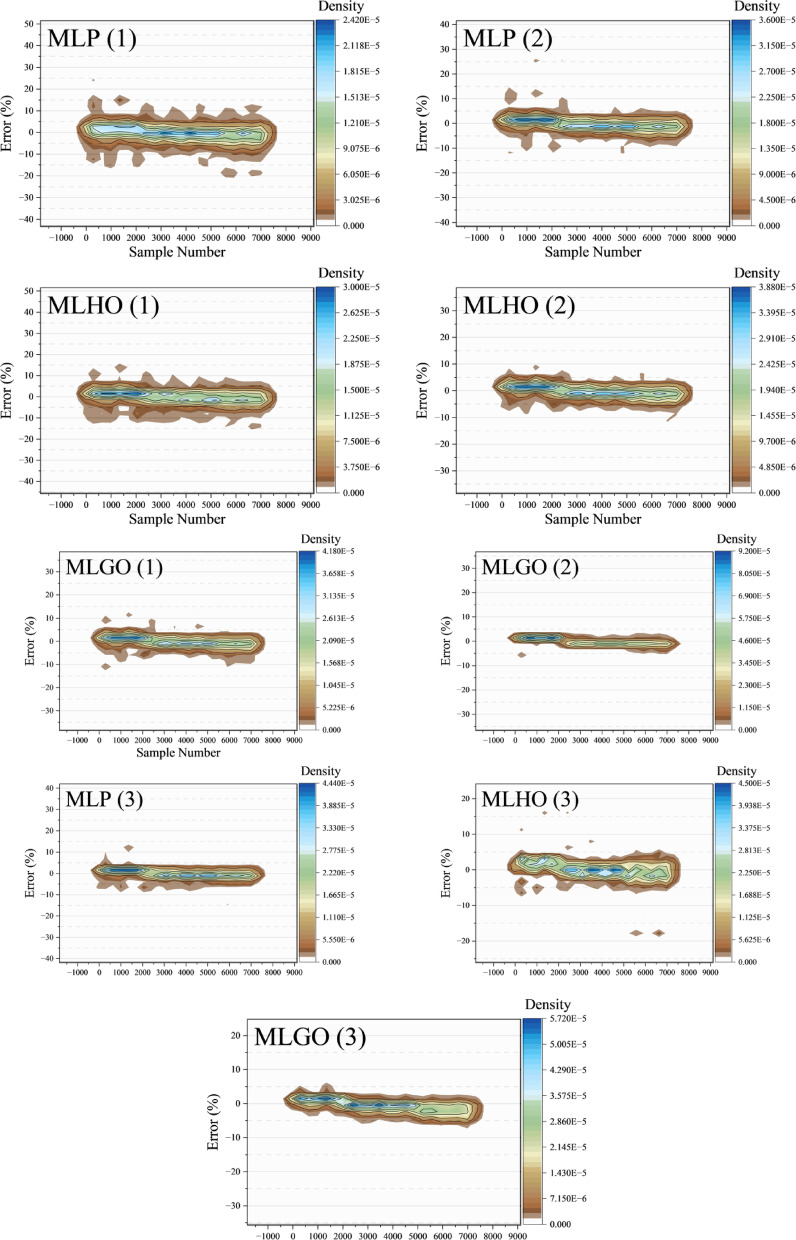
2D kernel density showing the progression of errors, illustrating how the model’s error changes over time.

Figure 5 illustrates 2D kernel density plots of the distribution of error percentages in models, showing how the error distribution varies with sample size and dataset points. The sample number is on the x-axis, representing the order of the dataset samples, and the percentage of error is on the y-axis, representing the distance from the true values in the predictions. The density gradient in the figure shows a distribution of error percentages, with darker points indicating higher density where errors occur more frequently. Figure 5 is consistent with the numerical results in showing that the distribution of the highest concentration is around 0% in the MLGO error (3), indicating lower prediction errors and better precision. The graph is consistent with the reported performance results, with the lowest RMSE (6.4780) and the highest R² (0.9881), indicating better predictive power. The MLHO (3) model shows a broader distribution of errors than in the MLGO (3) case, indicating greater variability in prediction precision, as reflected in the high RMSE (8.4012) and low R² (0.9773). The MLP (3) model shows the highest and most distributed errors, representing the least consistent predictions, with the highest RMSE (10.9639) and the lowest R² (0.9616) in Layer 3.

The performance results in Table 5 compare predictive precision and efficiency of diverse versions of the model in the train, validation, and test phases. The evaluation metrics are RMSE, R², MAPE, MARE, and TIC, with a comprehensive evaluation of every model’s ability. At every layer, the MLGO model outperforms the other models (MLP and MLHO) in predictive precision. In Layer 1, the least RMSE (11.5108) and highest R² (0.9576) are produced in the total evaluation phase in the MLGO. It reflects better generalization and lower prediction error. In a comparative performance with the traditional MLP model, better performance is in MLHO with lower RMSE (12.8863) and R² (0.9463), while in MLP, with lower RMSE (14.6320) and R² (0.9310). The performance is indicative of better MLGO performance, with optimized lower error and higher predictive precision. In Layer 2, performance is comparable to MLGO, with lower RMSE (8.2837) and the highest R² (0.9777) across the total phase. The performance is next with lower RMSE (10.4219) and R² (0.9655) in MLHO, while in MLP, with lower RMSE (12.0754) and R² (0.9536). Interestingly, the least MAPE (1.7330), MARE (0.0172), and TIC (0.0163) are produced in the MLGO, again in accordance with a better ability to achieve lower relative and absolute errors. In Layer 3, performance is still better in the MLGO, with lower RMSE (6.4780) and R² (0.9881), than in MLHO (RMSE: 8.4012, R²: 0.9773) and MLP (RMSE: 10.9639, R²: 0.9616). The improvement is even greater in the training phase, where MLGO achieves an RMSE of 4.8661 and an R² of 0.9923, demonstrating its ability to fit the data correctly while achieving superior generalization in the validation and test phases. The entire phase with a TIC value of 0.0128 also confirms its superior forecasting accuracy.

**Table 5:** Performance metrics of the MLP models, assessing their predictive accuracy and effectiveness using key statistical measures.

Layer	Models	Evaluation metrics					
		Phase	RMSE	R^2^	MAPE	MARE	TIC
(1)	MLP	Train	13.6619	0.9380	3.2240	0.0327	0.0269
		Validation	16.7836	0.9129	4.1236	0.0395	0.0334
		Test	16.5708	0.9333	4.0739	0.0386	0.0328
		Total	14.6320	0.9310	3.4864	0.0346	0.0289
	MLHO	Train	12.0375	0.9522	2.6623	0.0273	0.0237
		Validation	14.7312	0.9319	3.3106	0.0320	0.0292
		Test	14.6238	0.9482	3.3073	0.0315	0.0289
		Total	12.8863	0.9463	2.8563	0.0286	0.0254
	MLGO	Train	10.4876	0.9637	2.2397	0.0227	0.0206
		Validation	13.6109	0.9438	2.9396	0.0282	0.0270
		Test	13.5940	0.9566	2.9231	0.0276	0.0269
		Total	11.5108	0.9576	2.4472	0.0243	0.0227
(2)	MLP	Train	11.1000	0.9592	2.4162	0.0244	0.0219
		Validation	14.1834	0.9399	3.2513	0.0311	0.0282
		Test	13.9985	0.9549	3.1760	0.0300	0.0277
		Total	12.0754	0.9536	2.6554	0.0263	0.0238
	MLHO	Train	9.4748	0.9703	2.1180	0.0213	0.0187
		Validation	12.3131	0.9559	2.8713	0.0274	0.0245
		Test	12.3929	0.9649	2.8796	0.0272	0.0245
		Total	10.4219	0.9655	2.3452	0.0231	0.0206
	MLGO	Train	7.3365	0.9822	1.5522	0.0156	0.0144
		Validation	10.0457	0.9689	2.1119	0.0204	0.0199
		Test	10.2668	0.9728	2.1983	0.0210	0.0202
		Total	8.2837	0.9777	1.7330	0.0172	0.0163
(3)	MLP	Train	9.7884	0.9684	2.0547	0.0207	0.0193
		Validation	13.3340	0.9455	2.7520	0.0264	0.0265
		Test	13.2845	0.9580	2.7053	0.0255	0.0262
		Total	10.9639	0.9616	2.2569	0.0223	0.0216
	MLHO	Train	7.5112	0.9813	1.7649	0.0176	0.0148
		Validation	10.1368	0.9697	2.5489	0.0244	0.0201
		Test	10.2235	0.9734	2.4493	0.0234	0.0201
		Total	8.4012	0.9773	1.9851	0.0195	0.0166
	MLGO	Train	4.8661	0.9923	1.3683	0.0136	0.0096
		Validation	9.0962	0.9842	2.7438	0.0261	0.0181
		Test	9.3040	0.9852	2.7370	0.0260	0.0184
		Total	6.4780	0.9881	1.7799	0.0173	0.0128

**Fig. 6:**
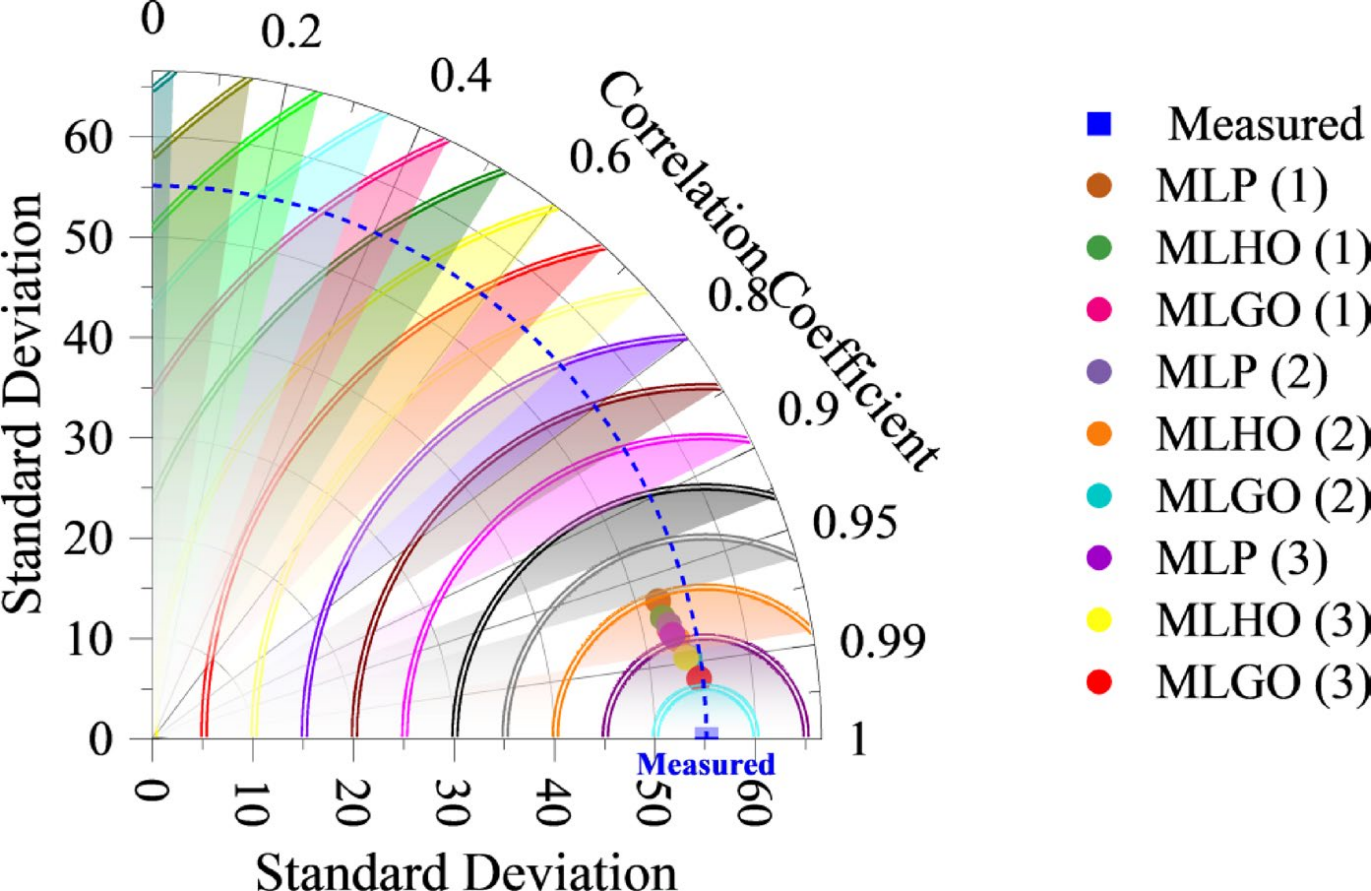
Taylor diagram showing the comparison of model performance with observed data.

Taylor diagrams (Fig. 6) are applied to graphically compare the performance of various models against a reference observation, which is CO2 emission. They illustrate three important statistical measures at once: the Correlation Coefficient (Radial Axis), which gauges the linear relationship between the model predictions and the observed data, ranging from 0 (no correlation) to 1 (perfect correlation); Standard Deviation (Radial Distance from Origin), which indicates the variability of the model predictions and the observed data, where the distance from the origin shows the magnitude of the standard deviation. The Measured data point is on the x-axis, which serves as the reference for comparison. Its location shows its standard deviation and serves as the origin for the RMSD arcs. Each colored point is a different model configuration (MLP, MLHO, MLGO) at different layers (1, 2, 3). The closer the model is to the “Measured” point, the better its performance, with higher correlation with the observed data, standard deviations closer to the observed data, and lower RMSD. The red point showing MLGO (3) is the closest to the “Measured” point, which means MLGO (3) has the highest correlation, a standard deviation very close to the measured data, and the lowest RMSD of all the models. This verifies that the GAO algorithm in the third layer of the MLP model gives the most accurate predictions.

To assess the statistical significance of model performance, a Wilcoxon signed-rank test was used to compare predicted CO₂ emissions with measured values across all evaluated models. The results are summarized in Table 6.

All models exhibit p-values well below the 0.05 significance threshold, confirming that the prediction errors are statistically meaningful rather than random. Among all configurations, MLGO (3) achieves the lowest Wilcoxon statistic and the highest rank, indicating the strongest agreement with measured emissions. This demonstrates that the GAO-optimized three-layer MLP significantly outperforms the remaining models in terms of distributional similarity to ground truth data. Although MLHO and standard MLP variants also show statistically significant performance, their Wilcoxon statistics and ranking positions are inferior to those of MLGO (3), confirming the advantage of GAO-based optimization combined with a deeper network architecture. These results provide formal statistical evidence supporting the superior predictive capability of the proposed MLGO (3) framework. The symbols “+” and “−” indicate statistically significant improvement, deterioration, or no significant difference, respectively, based on the Wilcoxon signed-rank test at a 0.05 significance level. Since all models exhibit p-values below 0.05, their predictions differ significantly from measured values. Among all configurations, MLGO (3) shows the strongest agreement with the ground truth, confirming its superior predictive performance.

**Table 6:** Result of the Wilcoxon test by comparing with measured values.

Models	Parameter				
	Statistical	*P*-value	Rank-*P*	Rank-stat	Significance
MLGO (1)	11,169,394	8.10E-33	6	7	+
MLHO (1)	12,445,434	1.28E-06	2	11	-
MLP (1)	10,940,711	8.53E-40	7	6	+
MLGO (2)	11,625,999	6.82E-21	4	9	+
MLHO (2)	10,404,378	6.77E-59	10	3	+
MLP (2)	10,110,098	9.48E-71	11	2	+
MLGO (3)	6,880,625	1.93E-279	12	1	+
MLHO (3)	12,642,362	0.000178	1	12	-
MLP (3)	10,826,620	2.51E-43	8	5	+

**Fig. 7:**
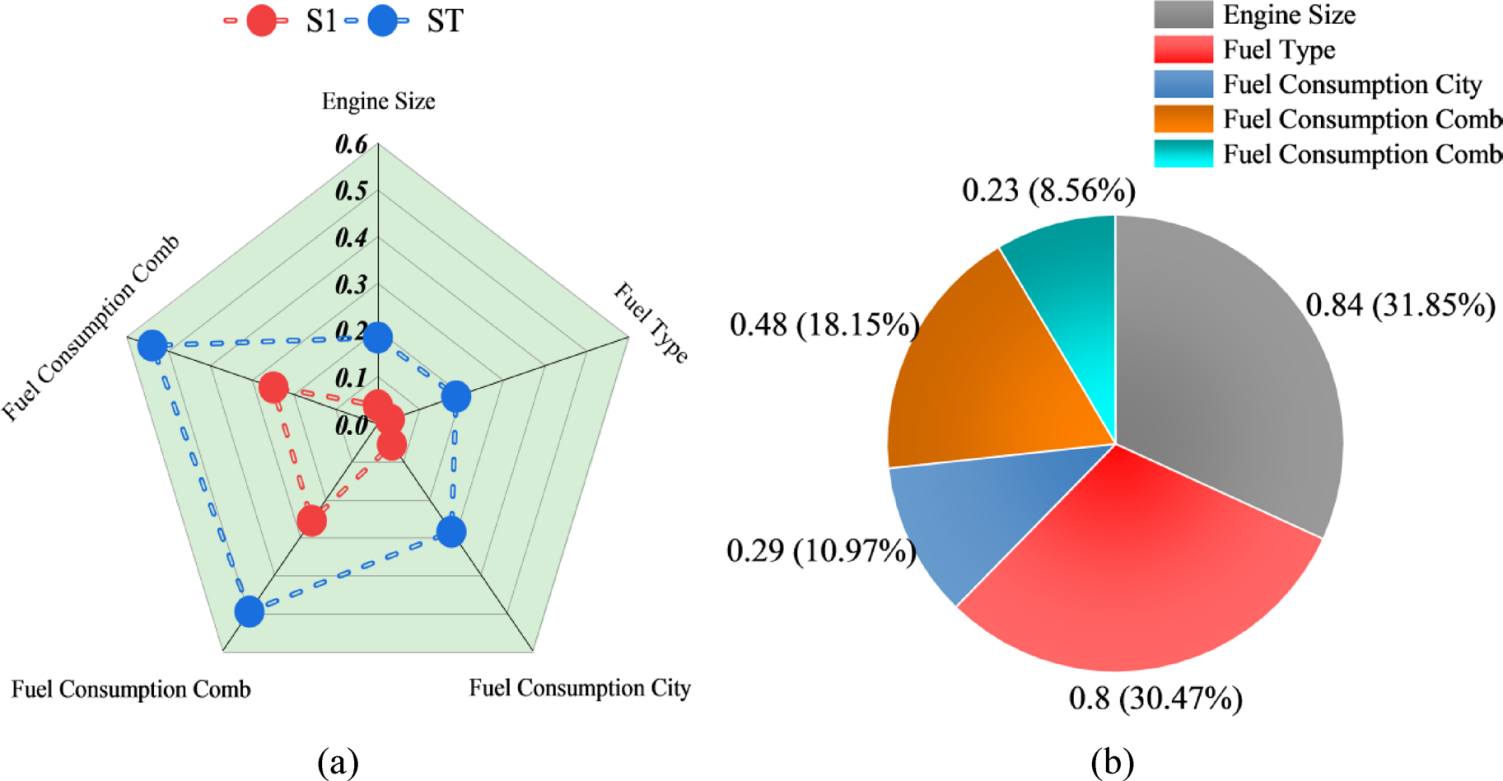
Sensitivity analysis conducted for the best-performing model (MLGO 3). (**a**) Radial plot for the CAM method, (**b**) pie chart illustrating the SHAP method.

Figure 7 illustrates the sensitivity analysis on the top-performing model in forecasting CO2 emissions identified in the research, the MLGO 3. It uses two methods in determining feature importance: Radial Plot (CAM Method) and Pie Chart (SHAP Method). The Radial Plot illustrates the model’s feature-sensitivity predictions using the Class Activation Mapping (CAM) method, where the radial axis shows the sensitivity (S1 and ST), and each spoke corresponds to a different input feature. The higher the value, the greater the sensitivity and the greater the contribution to the model’s predictions. The Pie Chart illustrates the contribution of each feature in the input using SHapley Additive exPlanations (SHAP), where each slice’s area represents the feature’s contribution to the model’s prediction, with numerical and percentage values indicating the contribution. Both the CAM and SHAP methods identify the features with the strongest influence as Engine Size and Fuel Consumption Comb (Combined Fuel Consumption).

These features are consistently ranked highest in both plots, in line with the findings from the RFE feature selection (Fig. 1) and the correlation matrix (Fig. 2), and also show that combined fuel consumption and engine size are important predictors of CO2 emissions. The SHAP pie chart clearly shows that Fuel Consumption Comb has the largest contribution (31.85%) to the model, in line with the high correlation observed in the correlation analysis. The second Fuel Consumption Comb is at 30.47%, and the combined fuel consumption levels contribute just over 60% to the model. Engine size is also ranked high in both plots, consistent with the correlation analysis, which showed a strong positive correlation between CO2 emissions and engine size. Fuel Type and Fuel Consumption City also contribute to the model’s predictions, but not to the same extent as combined fuel consumption and engine size. The RFE analysis is shown again, with Fuel Type ranking 3rd and Fuel Consumption City ranking 4th. The sensitivity analysis confirms the performance of the MLGO 3 model, shows how it relies on the most important features for predictions, and demonstrates that it is working correctly and has learned how the features correspond to the target variable. The radial plot in the CAM method shows the same information but in a different way, with the same trend in the strongest features, and the S1 and ST values representing the first-order and total effects of each feature.

**Fig. 8:**
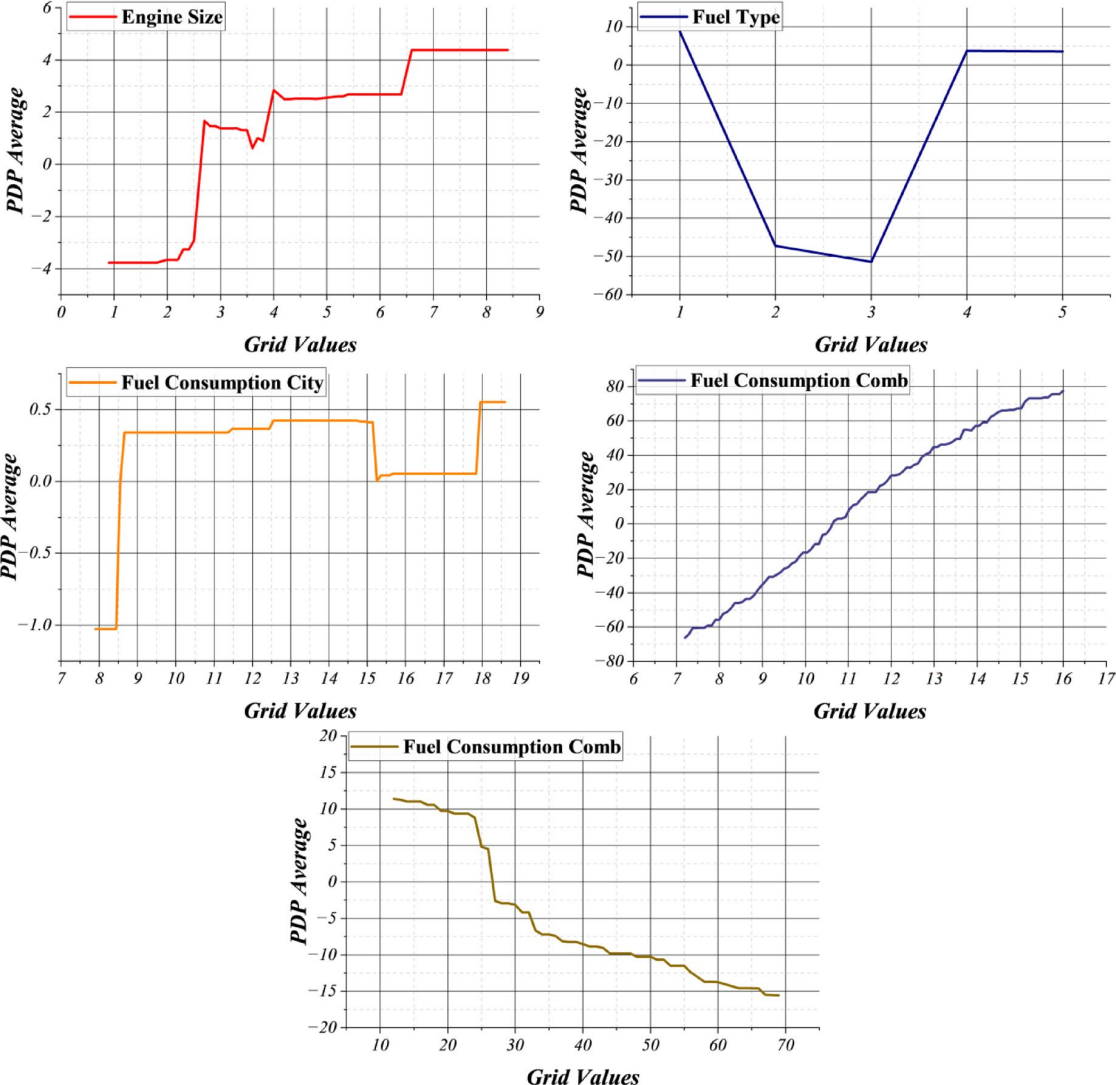
Result of the PDP values for the selected features.

Although several interpretability techniques exist, SHAP was adopted in this study because it provides both global and local explanations grounded in cooperative game theory, offering consistent, additive feature attributions across complex, nonlinear models. This enables direct quantification of each feature’s contribution while preserving theoretical guarantees of fairness and completeness. However, recognizing that MLPs primarily capture nonlinear relationships, SHAP was complemented with Partial Dependence Plots (PDPs) to visualize feature–response interactions explicitly. As shown in Fig. 8, PDP analysis reveals strong nonlinear dependencies between CO₂ emissions and key predictors such as engine size and fuel consumption. In particular, combined fuel consumption exhibits a monotonic yet nonlinear increase in emission response, while engine size shows threshold-like behavior, indicating regime changes in emission growth. Fuel type and urban fuel consumption also display non-uniform impacts across their value ranges. By integrating SHAP for quantitative importance ranking with PDP for nonlinear functional interpretation, the proposed framework provides both contribution-based and behavior-based explanations. This dual interpretability strategy ensures transparent assessment of feature influence while uncovering complex emission dynamics captured by the MLP model.

### Theory-constrained neural networks and physics-guided extensions

The present emission modeling framework adopts a data-driven learning paradigm in which the MLP parameters are optimized using metaheuristic algorithms (HLOA and GAO) to minimize prediction error. While this strategy demonstrates strong predictive accuracy, it does not explicitly embed physical or theoretical constraints governing vehicle energy conversion and emission generation. Consequently, the learned mappings rely primarily on statistical correlations within the training data. Recently, theory-constrained neural networks (TCNNs) and physics-informed learning paradigms have emerged as powerful alternatives that integrate domain knowledge directly into neural architectures or loss functions^35^. In such approaches, governing equations, conservation laws, or system-level constraints are introduced as soft penalties or modular components to guide model training toward physically consistent solutions. For vehicle and powertrain applications, this may include constraints on energy balance, drivetrain losses, combustion efficiency, and thermodynamic relationships.

For example, modular theory-constrained neural network frameworks have been proposed for fuel cell vehicle modeling, where physical submodules representing electrochemical processes and energy flow are coupled with neural networks to preserve interpretability and improve extrapolation capability. These models demonstrate that embedding theoretical structure not only enhances robustness under unseen operating conditions but also enables clearer attribution of system-level behavior to individual physical components. In the context of CO₂ emission prediction, similar constraints could be incorporated by:

- Enforcing energy conservation between fuel input, engine output, drivetrain losses, and vehicle traction demand.
- Introducing physics-based regularization terms related to fuel consumption–CO₂ proportionality.
- Structuring the network into interpretable modules (e.g., engine, transmission, aftertreatment) with parameter sharing.
- Penalizing physically implausible predictions during training.

Such theory-guided formulations are expected to reduce overfitting, improve generalization across vehicle platforms, and enhance interpretability beyond post-hoc explanation methods such as SHAP and CAM. Although the current study focuses on metaheuristic-optimized MLPs with explainable feature attribution, integrating theory-constrained learning represents a promising future research direction. Combining TCNNs with population-based optimizers such as GAO and HLOA could further improve convergence toward physically meaningful solutions while maintaining high predictive accuracy, particularly when labeled data are limited or operating conditions extend beyond the training domain. Accordingly, future work will explore hybrid physics–data-driven architectures that unify metaheuristic optimization with embedded theoretical constraints, following recent advances in modular and theory-constrained neural network modeling for vehicle systems.

### Vehicle-type–wise error characteristics

To further investigate model behavior, prediction errors were analyzed across different vehicle categories based on fuel type and powertrain characteristics. The largest prediction deviations were observed for vehicles with higher engine displacement and multi-cylinder configurations, particularly those representing performance-oriented or less frequent vehicle classes in the dataset. This behavior can be attributed to two main factors. First, these vehicle types exhibit stronger nonlinear interactions among engine, transmission, and fuel consumption variables, increasing modeling complexity. Second, they are comparatively underrepresented in the dataset, which limits the ability to learn stable emission patterns.

In contrast, compact and mid-range vehicles with conventional configurations demonstrated lower prediction errors due to their more homogeneous operating characteristics and higher sample density. These results indicate that prediction performance is influenced not only by model capacity but also by data distribution and mechanical variability. Future work will investigate class-balanced training and physics-informed constraints to improve robustness for high-variance vehicle categories further.

### Advantages, limitations, and future research directions

#### Advantages of the proposed framework

The proposed GAO/HLOA–MLP framework demonstrates several advantages. First, integrating metaheuristic optimization with deep neural networks enables effective exploration of complex hyperparameter spaces, resulting in improved convergence stability and prediction accuracy. Second, the combination of SHAP and CAM provides complementary global and local interpretability, enhancing the transparency and trustworthiness of the emission predictions. Third, the multi-modal feature engineering pipeline, incorporating correlation analysis and RFE, ensures that physically meaningful variables dominate the learning process. Finally, the framework is scalable and can be readily integrated with intelligent transportation systems for fleet-level emission monitoring.

#### Limitations

Despite these strengths, several limitations remain. The current study relies on offline training and static datasets, which may not fully capture evolving vehicle behaviors or real-time traffic dynamics. The dataset does not explicitly include transmission efficiency or exhaust aftertreatment performance, which could further improve the accuracy of emission estimates. Moreover, although GAO and HLOA improve optimization robustness, metaheuristic methods inherently incur higher computational costs than gradient-based training. In addition, explicit physical constraints were not embedded directly into the neural network architecture.

#### Future research directions

Future work will focus on extending the framework toward real-time deployment by integrating streaming telemetry from intelligent transportation systems. Theory-constrained neural networks will be investigated to embed physical emission bounds and powertrain principles directly into the learning process. Reinforcement learning–based control strategies will also be explored for closed-loop emission reduction and energy management. Furthermore, hybrid optimization schemes combining GAO and HLOA in collaborative architectures will be examined to accelerate convergence. Finally, incorporating additional powertrain descriptors, such as transmission efficiency and exhaust aftertreatment effectiveness, along with time-aware validation strategies, will further enhance robustness for practical applications.

## Conclusion

The results of this research highlight substantial improvements in CO₂ emissions prediction achieved by combining a three-layered Multi-Layer Perceptron (MLP) model with state-of-the-art metaheuristic optimization algorithms. Utilizing the Horned Lizard Optimization Algorithm (HLOA) and the Giant Armadillo Optimization (GAO), the research has effectively improved prediction accuracy and convergence efficiency. The superior performance of the GAO-optimized model (MLGO) at each layer, especially in Layer 3, indicates the effectiveness of metaheuristic algorithms in optimizing predictive models. The MLGO 3 recorded the highest R² value of 0.9881 and the lowest RMSE of 6.4780, verifying its superior ability to reduce prediction error and enhance generalization across the training, validation, and testing phases. One of the major contributions of this research is the utilization of Recursive Feature Elimination (RFE) in conjunction with sensitivity analyses using SHapley Additive Explanations (SHAP) and Class Activation Mapping (CAM). These tools provided vital insights into the most influential predictors of CO₂ emissions, with engine size and combined fuel consumption emerging as the dominant determinants. The SHAP analysis indicated that fuel consumption factors accounted for more than 60% of the predictive model’s accuracy, underscoring the role of vehicle efficiency in emission control measures. This degree of interpretability improves the model’s applicability in real-world settings, making it a useful tool for policymakers, urban planners, and automotive manufacturers. The convergence analysis also verified the robustness of the optimization process. The decreasing trend in convergence values demonstrates that the model progressively improves its weight and bias parameters, resulting in an optimized solution. The overall superiority of the MLGO model on a variety of evaluation criteria, including RMSE, MAPE, MARE, and TIC, confirms the efficiency of integrating GAO with the MLP paradigm. It shows the promise of hybrid machine learning approaches in capturing complex, nonlinear relations in environmental forecasting. From a practical standpoint, the implications are vast. By offering a consistent, highly accurate prediction model for CO₂ emissions, the research provides a sound decision-support system for a wide array of stakeholders. Automotive manufacturers can leverage these insights to design efficient, low-emission vehicles, while policymakers can design policies grounded in facts to address environmental impacts. Furthermore, these predictive models can be used to calculate the ecological load of vehicle traffic and to design policies accordingly. Overall, the research is well-positioned to successfully close a significant knowledge gap in forecasting CO₂ emissions by integrating advanced machine learning with state-of-the-art optimization. The improved performance of the GAO-optimized MLP model makes it a valuable predictive tool in green transportation planning. The following research could be explored in greater detail to integrate with other optimization techniques or to augment the dataset with real-time vehicle emissions monitoring. The research opens the way for better, fact-driven approaches in order to address global warming and incentivize environmentally friendly modes of transport.

## Supplementary Information

Below is the link to the electronic supplementary material.

Supplementary Material 1

## Data Availability

To enhance transparency and reproducibility, all model implementations, hyperparameter configurations, trained weights, and calculation results have been provided as supplementary material in a compressed ZIP file. This package includes the complete source code for GAO–MLP and HLOA–MLP models, preprocessing scripts, and result files required to reproduce the experiments reported in this study.
